# A Digital-Twin Framework for Predicting the Remaining Useful Life of Piezoelectric Vibration Sensors with Sensitivity Degradation Modeling

**DOI:** 10.3390/s23198173

**Published:** 2023-09-29

**Authors:** Chengcheng Fu, Cheng Gao, Weifang Zhang

**Affiliations:** 1 School of Energy and Power Engineering, Beihang University, Beijing 100191, China; iamfcc@buaa.edu.cn; 2 School of Reliability and Systems Engineering, Beihang University, Beijing 100191, China; zhangweifang@buaa.edu.cn

**Keywords:** digital-twin framework, remaining useful life prediction, piezoelectric vibration sensor, sensitivity degradation, accelerated degradation model

## Abstract

Piezoelectric vibration sensors (PVSs) are widely applied to vibration detection in aerospace engines due to their small size, high sensitivity, and high-temperature resistance. The precise prediction of their remaining useful life (RUL) under high temperatures is crucial for their maintenance. Notably, digital twins (DTs) provide enormous data from both physical structures and virtual models, which have potential in RUL predictions. Therefore, this work establishes a DT framework containing six modules for sensitivity degradation detection and assessment on the foundation of a five-dimensional DT model. In line with the sensitivity degradation mechanism at high temperatures, a DT-based RUL prediction was performed. Specifically, the PVS sensitivity degradation was described by the Wiener–Arrhenius accelerated degradation model based on the acceleration factor constant principle. Next, an error correction method for the degradation model was proposed using real-time data. Moreover, parameter updates were conducted using a Bayesian method, based on which the RUL was predicted using the first hitting time. Extensive experiments on distinguishing PVS samples demonstrate that our model achieves satisfying performance, which significantly reduces the prediction error to 8 h. A case study was also conducted to provide high RUL prediction accuracy, which further validates the effectiveness of our model in practical use.

## 1. Introduction

Aeroengines for aircraft must balance requirements of high power, lightweight, safety, reliability, and continuity of service [1]. While working in harsh environments, the high-speed rotation inevitably causes vibration and, thus, component degradation. Therefore, detecting and reducing vibration are highlighted. Nevertheless, considering the complexity of internal combustion, the elimination of vibration based on the working principle of aeroengines remains challenging [2]. Therefore, only when the vibration of an aeroengine is measured in real-time can the safety of an aircraft be maintained to the greatest extent [3]. The exploitation of piezoelectric vibration sensors (PVSs) provides one approach to this challenge, due to the stability of their long-term operation under high-temperature conditions [4]. As an example, the CFM56 turbofan engine in the Boeing 737 uses PVSs to identify working states in every individual rotational cycle [5]. Studies are ongoing that aim to develop PVS-based methods for aeroengine condition monitoring.

PVSs are highly significant, and improvements in their reliability are paramount [6,7]. Despite advances in the optimization of their materials and structure, the risk of PVS failure due to the effect of thermo-mechanical coupling, restricted to aeroengine working conditions, still exists [8,9]. As a result, remaining useful life (RUL) prediction, on the basis of PVS degradation assessments, was proposed [10]. Current RUL prediction approaches are generally carried out based on either physical failure patterns or data-driven models. With respect to the degradation of PVSs, they have two main limitations. First, the stress distribution in working PVSs is so complex that the application of the physical failure model fails to perform its working state precisely [11]. Second, in line with the confidentiality principle, the onboard data for PVS malfunction characterization is currently inaccessible. For this reason, accurate data-driven PVS degradation modeling remains absent.

More recently, advances in DT gave rise to opportunities to develop RUL prediction methods. Theoretically, a DT is a digital representation of a physical structure that comprises the selected elements and dynamics within its lifespan, which bridges the gap between the virtual and the real world [12,13]. Based on real-time interactions between virtual models and physical structures, the degradation of a target system, especially a sensing device, can be derived and evaluated effectively. Recently, Feng et al. developed a DT-driven intelligent method to assess gear surface degradation under harsh conditions [14]. Jafari and Khadim et al. devised a DT-based state-estimation method to model the dynamics of hydraulically actuated machinery [15]. Byun proposed a DT framework for battery-state prediction, which improves the accuracy of battery management [16]. Don et al. used vibration data for the fatigue-life prognosis of a vertical oil well drill string [17]. Accordingly, by modeling the mechanical structure, working principle, and degradation mechanism, a DT methodology is capable of digitizing complicated electromechanical equipment [18]. Despite the complex working conditions of PVSs, a DT solution can be applied to RUL predictions.

The use of DT in PVS degradation models is, however, still limited because of the primary challenge of establishing high-fidelity physical and virtual twins. Each PVS, which consists of multiple components, has multiple properties that vary under different conditions. A DT model cannot be built until synchrony between the physical structure and the digital model is achieved. Furthermore, an accurate RUL prediction approach is also expected. The operational process inevitably deteriorates the status of a PVS, and the available information has to be specifically adapted and used to complete the degradation assessment.

The objective of this work is to propose a PVS-specific DT method for the task of RUL prediction. We focus here on establishing a precise DT model to characterize the environment and state of a PVS. Our method aims to predict the RUL in a novel way by using the data of both working and testing conditions.

The contribution of this work is threefold and summarized as follows:

A novel DT framework is designed and deployed based on the working principle of PVSs. The scheme paves the way for DT-based modeling of similar devices.

A RUL prediction is performed using both historical degradation data and real-time degradation data, and provides accurate RUL prediction results for the PVS.

Experiments are conducted to validate the working performance on a set of PVS samples. The experimental results show that our method is superior in RUL prediction tasks under different conditions.

## 2. Prerequisite

### 2.1. PVS-Specific DT

**Figure 1:**
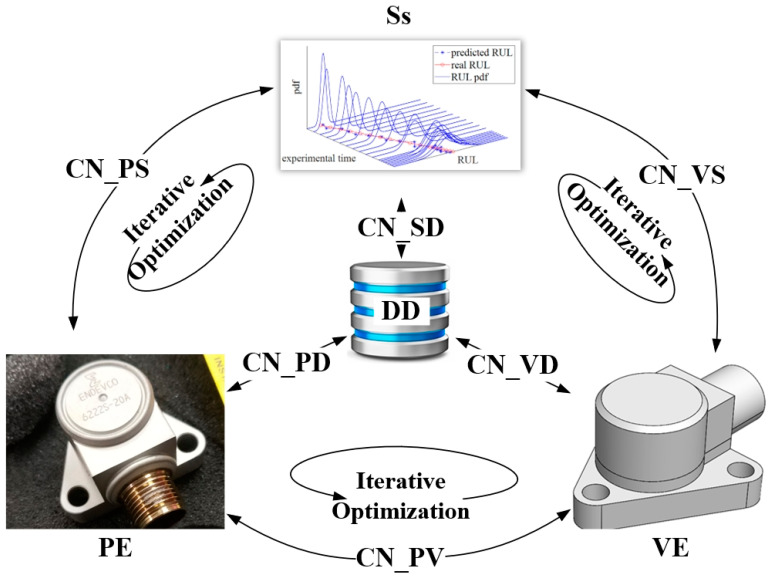
Architecture of five-dimension DT model.

During the task of vibration detection [19], a PVS is capable of converting vibrations into an electrical signal via the piezoelectric effect. Similar to a collection of sensing devices [20,21,22], a PVS has a complex structure and harsh working conditions. In this work, a PVS-specific DT framework is established based on a five-dimensional DT architecture. The standard five-dimensional DT model was originally proposed by Tao et al. for the description of complex systems [23,24]. The architecture of a typical five-dimension DT model is presented in Figure 1. A five-dimensional DT model covers not only the physical entities, virtual entities, and connections between them, but also the data and services; see Equation (1) [25].

(1) $$M_{DT}=\left(PE,\;VE,Ss,DD,CN\right)$$

where $M_{DT}$ is the five-dimension DT model; PE represents the physical entity; VE is the virtual equipment; Ss indicates the services for *PE* and *VE*; DD refers to DT data; and CN is the connection among other parts.

More details of the PVS-specific DT model are presented below.

#### 2.1.1. Physical Entity (PE)

**Figure 2:**
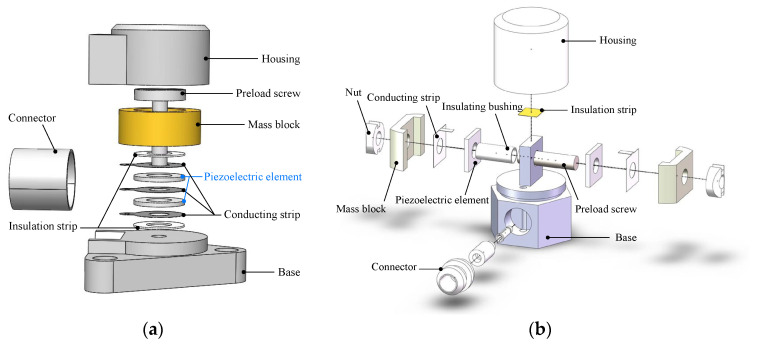
The main structure types of PVSs: (**a**) shear-type PVS and (**b**) compression-type PVS.

The *PE* of the PVS-specific DT model contains the body of the PVS, its components, and the attached measuring devices [23]. Generally, the PVS can be categorized as a shear type and a compression type, which are respectively subjected to shear stress and compressive stress. Figure 2 shows the structure of both types, which consist of similar components but have distinguishing mechanical characteristics. For both types, a PVS is composed of a casing, a base, a connector, two mass blocks, a pre-load screw-pair, two piezoelectric strips, two conducting strips, and an insulating strip. The pre-load is applied to the mass block, the piezoelectric strip, and the conducting strip, which are assembled with the pre-load screw-pair. Notably, the pre-load direction of the compression-type PVS is consistent with the sensitive direction, while that of the shear type is vertical to the sensitive direction. In addition, the measuring device is used for the PVS test, which contains a charge amplifier, signal acquisition equipment, calibration equipment, and temperature sensing elements.

#### 2.1.2. Virtual Equipment (VE)

The *VE* is a high-fidelity digital model of *PE*, which reproduces geometries, physical properties, behaviors, and rules of *PE* in the virtual world [23]. In this work, the *VE* of a PVS is subdivided into three models as follows

(2) $$VE=\left(M_{signal},M_{simu},M_{de}\right)$$

where $M_{signal}$ is the vibration–electrical conversion model, $M_{simu}$ is the simulation model for mechanical and thermal stress characterization, and $M_{de}$ is the RUL prediction model.

**Vibration–Electrical Conversion Model** $M_{signal}$: The critical function of a PVS is vibration–electrical signal conversion. We use sensitivity to describe the relationship between output and input, as presented in Equation (3).

(3) $$S=\frac{q\left(t\right)}{a(t)}$$

where S represents the PVS sensitivity with the dimension of $\left(\mu C\cdot m^{-1}\cdot s^{2}\right)$, $q\left(t\right)$ is the output charge (μC) at time t, and a(t) is the input vibrating acceleration ($m\cdot s^{-2}$) at time t.

Precisely, the vibration is sensed via charge signals and converted into electrical voltages using a charge amplifier. Then, with filters applied, a sinusoidal waveform is obtained.

(4) $$v_{hp}\left(t\right)=k\cdot q\left(t\right)$$

where $v_{hp}\left(t\right)$ is the half peak of the output voltage (V) at time t and k is the conversion factor of the charge amplifier (V/μC).

Substituting Equation (3) into Equation (4), the vibrating acceleration is computed as:

(5) $$a\left(t\right)=\frac{v_{hp}\left(t\right)}{k\cdot S}$$

Aiming at measuring the PVS sensitivity, a set of vibration signals are sent to two PVSs, i.e., a measurement standard PVS and a target PVS. According to Equation (5), for both PVSs, we have:

(6) $$a\left(t\right)=\frac{v_{hp1}\left(t\right)}{k_{1}\cdot S_{1}}=\frac{v_{hp2}\left(t\right)}{k_{2}\cdot S_{2}}$$

which can be transformed into:

(7) $$S_{2}=S_{1}\cdot \frac{k_{1}}{k_{2}}\cdot \frac{v_{hp2}\left(t\right)}{v_{hp1}\left(t\right)}$$

where $S_{1}$, $k_{1}$, and $v_{hp1}\left(t\right)$ are parameters of the measurement standard, while $k_{2}$ and $v_{hp2}\left(t\right)$ can be measured during testing. The sensitivity of the target PVS is thus determined.

**Simulation Model** $M_{simu}$ with simplified structure: As pointed out in the Introduction, the working environment of a PVS is severe, which results in complex mechanical and thermal stress. The simulation model is performed using finite element analysis (FEA) to reproduce the working status of a PVS. The natural frequency is exploited to demonstrate the vibration property. Notably, the simulation model is built based on structure modeling. We shall thus investigate the significance of each component and simplify the structure of PVS to facilitate the simulation.

**Figure 3:**
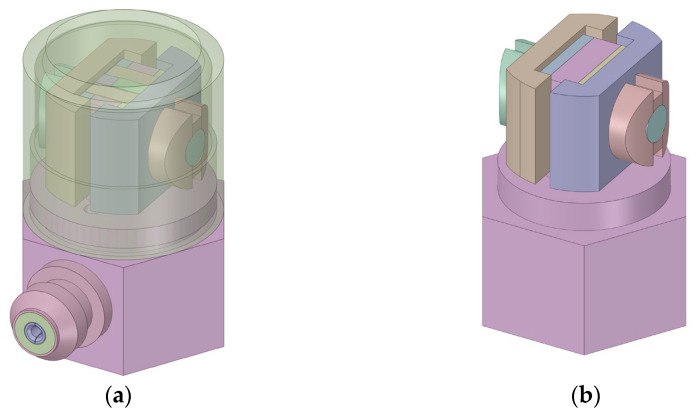
Structure of a shear-type PVS used in the simulation model: (**a**) original and (**b**) simplified.

**Figure 4:**
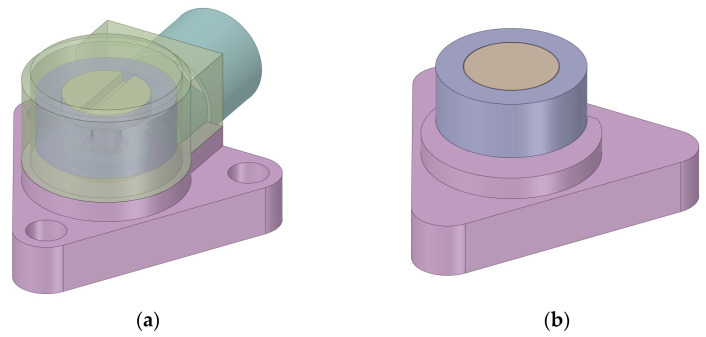
Structure of the compression PVS used in the simulation model: (**a**) original and (**b**) simplified.

Using the simulation analysis shown in Appendix A, the simplified structure of a shear-type PVS is derived and presented in Figure 3. A comparison between the simplified structure and the original compression PVS is given in Figure 4.

**Figure 5:**
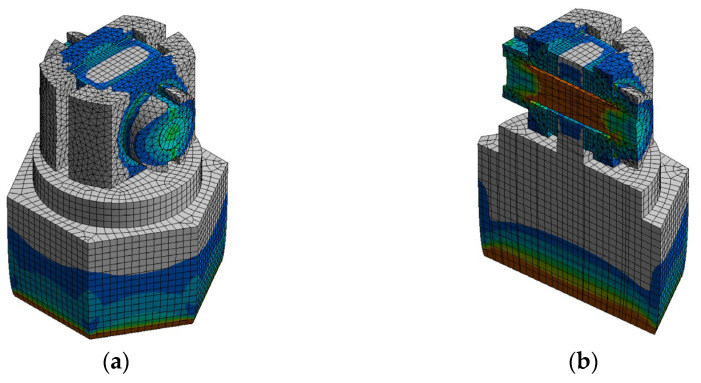
Stress distribution simulation results for shear PVS: (**a**) overall view and (**b**) sectional view.

**Figure 6:**
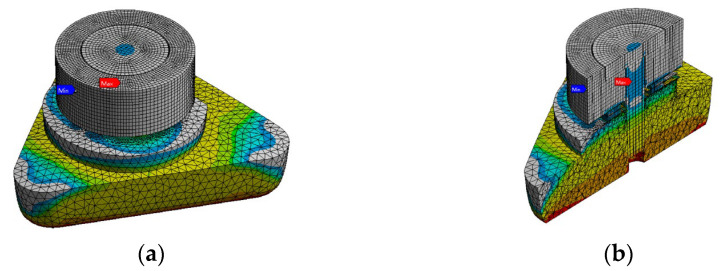
Stress distribution simulation results for compression PVS: (**a**) overall view and (**b**) sectional view.

Using a simplified structure, a more efficient stress model is thus built for simulation. With respect to natural-frequency-based simulation, primary attention is paid to the stress simulation of piezoelectric elements. Considering that the stress of piezoelectric sheets is transmitted from the pre-load structure to its surfaces, we perform a more fine-grained meshing on the contact surfaces, which aims to maintain consistency on both sides. Furthermore, considering the working environment and the PVS thermal conductivity, the simulation temperature is set within the range of 423.15 K and 493.15 K. Examples of simulation outcomes for both shear PVS and compression PVS at the temperature of 473.15 K are presented in Figure 5 and Figure 6.

**RUL Prediction Model** $M_{de}$: The RUL prediction model is composed of a degradation unit, an accelerated degradation unit, and a life prediction unit.

The degradation of PVS is characterized by a stochastic process. For one thing, sensitivity degradation is performed in a non-monotonic and non-linear fashion, which is affected by thermal stresses and varies for every single PVS. For another, in the context of PVS RUL prediction, a large amount of sensitivity degradation data is unavailable, especially for consistent working conditions. For this reason, a stochastic process, with remarkable statistical properties and mathematical fitting, is used not just to describe a non-monotonic and non-linear trend but also to address the issue of a small sample [26,27]. A non-linear Wiener stochastic process is used to describe PVS sensitivity degradation.

Furthermore, accelerated degradation denotes the relation between performance deterioration and applied stress. Following the idea of [28], accelerated degradation carried out in a stochastic process is capable of resolving inconsistent degradation under different thermal stress conditions. The Arrhenius model, which relates to thermal stress modeling, is used. The acceleration factor constant principle (AFCP) is applied to the accelerated degradation modeling design and parameter determination. In such a manner, a Wiener–Arrhenius sensitivity accelerated degradation process is also developed.

Parameters of both units can be finetuned in line with real-time sensitivity degradation with PVS calibration. All degradation data are analyzed to obtain a degradation mechanism, which is applied to PVS RUL prediction. The degradation data generation and RUL prediction are conducted in the framework of Python 3.9.0. The approaches for each process are depicted in detail in Section 4.

#### 2.1.3. Services (Ss)

Following the idea of Tao et al., *Ss* involves the optimization of *PE* whilst adjusting *VE* to sustain its performance with *PE* [23]. With respect to the PVS-specific DT model, *Ss* contains external device control and operation, model management and application, data storage and processing, visualization, etc.

#### 2.1.4. DT Data (DD)

*DD* contain the data from *PE*, *VE*, and *Ss* domain-specific knowledge as well as their fusion [23]. *PE* data concerns data on design, operation, and testing. More specifically, test data come from both historical tests and real-time tests of PVS. *VE* data relate to the model establishment, simulation, and computation. *Ss* data are collected during all service procedures. The fusion data is the integration of all the above data.

#### 2.1.5. Connections (CNs)

A *CN* is devised for the interconnection of all other parts, based on which six bidirectional connections, i.e., *Ss* and *DD*, *PE* and *DD*, *VE* and *DD*, *PE* and *Ss*, *VE* and *Ss*, and *PE* and *VE*, are respectively built [23]. In RUL prediction, the connection between *PE* and *DD* sets a foundation for state monitoring. The interaction among various connecting parts is utilized to recall historical PVS data, identify current degradation, and obtain real-time processing results. In certain cases, human intervention is also involved with the connection, especially for *PE* adjustment and system initialization.

### 2.2. Failure Modes of PVS

An abnormality that characterizes sensor failure is abnormal output, which is also true for the failure of a PVS. A vibration is delivered from the base to the pre-loaded structure and further reaches the piezoelectric strip. The electrical charges are thus generated and sent out via a signal transmission wire. In this way, a sensor malfunction results wherever a problem arises. In general, failure modes of PVS contain structure damage, electrical faults, and sensitivity out of tolerance. Currently, the widely used PVSs are made of stainless steel and tungsten of high rigidity and stability, which are capable of resisting mechanical destruction. Moreover, the reliability of electrical components can be established with design assurance and processing assurance. By contrast, sensitivity degradation plays a pivotal role in RUL prediction results. Considering that a PVS is a piezoelectric-based sensing device, piezoelectric crystals tend to degrade under the condition of intensive stress. Recent publications reveal crack diffusion and phase transitions of piezoelectric elements caused by stress application [29,30,31]. Furthermore, owing to the disparity in the thermal expansion coefficient, a high environmental temperature also leads to a stress increase in a PVS.

As an example, a shear-type PVS works under high-temperature conditions for hundreds of hours, which results in sensitivity out of tolerance. After disassembling components, cracks on a piezoelectric strip are observed. Either a piezoelectric element malfunction or a pre-load manner can induce this failure. For this reason, the sensitivity has to be calibrated in time, based on which the degradation trend can be documented for working state analysis.

## 3. Methodology

**Figure 7:**
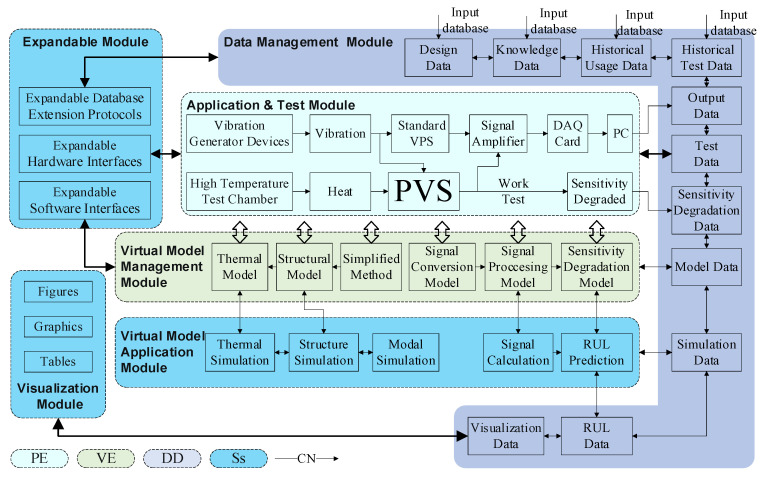
The architecture of a DT for RUL prediction.

Aiming at predicting the RUL of PVS, a DT-driven sensitivity degradation assessment framework is established. On the one hand, this framework involves all five dimensions in the proposed PVS-specific DT model. On the other hand, the RUL prediction method has six basic modules that present the real-time PVS status, deal with both physical and virtual working conditions, diagnose sensitivity degradation, and provide users with prediction results. The architecture of the proposed method is shown in Figure 7. Each module in this method is described in detail below.

**Application and test module**: The application and test module mainly contain the *PE* setup for PVS working and testing and the *VE* application for modeling and simulation, together with *Ss*, *DD*, and *CN* generated and utilized during operation. The PVS sensitivity measurement is carried out in the application and test module. This module concerns a target PVS, measurement devices, environmental devices, and the sensitivity degradation test scheme. For the PVS test, a measurement standard, a charge amplifier, a DAQ card, and a host computer are used as measurement devices. In addition, the environmental devices consist of a vibration exciter, a power amplifier, a waveform generator for vibration signal generation, and a high-temperature chamber for simulating severe working environments.

**Virtual model management module**: The virtual model refers to all models in *VE*, i.e., a vibration–electrical conversion model, a simulation model, and a degradation model. The virtual model management module is designed as an auxiliary to the application and test modules, which provides a more accurate description of *PE* in virtual space for RUL prediction.

**Virtual model application module**: This module relates to *Ss* and *CN* and bridges the gap among *PE*, *VE*, and *DD*. Specifically, not only the thermal, structural, and modal simulations but also the data computations are incorporated in the virtual model application module. More details of the RUL prediction algorithm are presented in Section 4.

**Data management module**: The data management module supports the interactions among different modules. In the proposed method, the measurement and sensitivity degradation data are collected in the application and test modules; the model information is conveyed from the virtual model management module, and the simulation and prediction results are generated in the virtual model application module. All data are stored in the database of *DD*.

**Expandable module**: The expandable module is responsible for the expansion of *PE* devices, *VE* models, Ss protocols, and *DD* datasets. The method upgrade is also performed in this module.

**Figure 8:**
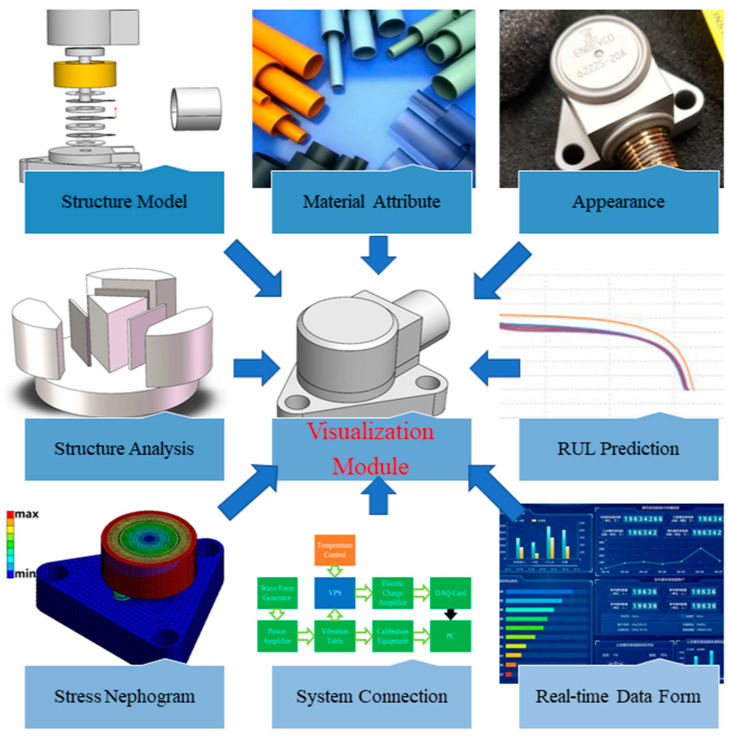
Interface of the visualization module.

**Visualization module**: The visualization module is one branch of *Ss* that illustrates the information of each module. The interface of the visualization module is shown in Figure 8.

## 4. RUL Prediction Algorithm

In the virtual model application module, two processing steps can be distinguished when performing RUL prediction: sensitivity degradation modeling and life prediction. Specifically, sensitivity degradation is further characterized by the acceleration factor constant principle (AFCP), while the life prediction is conducted using a dynamic correction mechanism.

### 4.1. Sensitivity Degradation Modeling

From the description above, PVS sensitivity degradation is generally influenced by thermal stresses under working conditions, which is defined as accelerated degradation. To start with, considering the randomness and individual differences in each sensing device, sensitivity degradation is performed in a stochastic process. A non-linear Wiener process is used to model non-monotonical and non-linear degradation data [27]. First, define the sensitivity degradation at time t as:

(8) $$x\left(t\right)=x\left(0\right)+\lambda \Lambda \left(t\right)+\sigma B\left(\Lambda \left(t\right)\right)$$

and

(9) $$x\left(0\right)=0$$

With a power–time function $\Lambda \left(t\right)=t^{\eta }$, the stochastic sensitivity degradation process is transformed to:

(10) $$x\left(t\right)=\lambda t^{\eta }+\sigma B\left(t^{\eta }\right)$$

where *λ* is a drift coefficient that denotes individual differences among the same batch samples; σ refers to the diffusion coefficient; $B\left(\cdot \right)$ is standard Brownian motion, and η is used for time scaling. For each $∆x\left(t\right)=x\left(t+∆t\right)-x\left(t\right)$, the $∆x\left(t\right)$ correlates to the normal distribution, i.e., $∆x\left(t\right)\sim N\left(\lambda ∆\Lambda \left(t\right),\sigma^{2}∆\Lambda \left(t\right)\right)$.

With respect to the PVS in this work, degradation data are obtained from an accelerated degradation test. Let $x_{ijk}$ be the sensitivity degradation of the j-th sample at the i-th time under stress $T_{k}$, with $t_{ijk}$ representing the specific observation time. We respectively derive the sensitivity degradation increment and the time increment as:

(11) $$∆x_{ijk}=x_{ijk}-x_{(i-1)jk}$$

and

(12) $$\Lambda_{ijk}=t_{ijk}^{\eta }-t_{(i-1)jk}^{\eta }$$

where k∈[1,M], $j\in \left[1,N_{k}\right],$ and $i\in [1,H_{jk}]$.

For $∆x_{ijk}\sim N\left(\lambda_{jk}∆\Lambda \left(t\right),\sigma_{jk}^{2}∆\Lambda \left(t\right)\right)$, the parameter set Θ=[σ,λ,η] can be determined using maximum likelihood estimation (MLE). The estimation data are collected from the historical sensitivity degradation data on the PVS under the same working conditions. The likelihood function of MLE is written as:

(13) $$L_{jk}\left(\theta \right)=\prod_{i=1}^{H_{jk}}\frac{1}{\sqrt{2\pi \sigma_{jk}^{2}∆\Lambda_{ijk}}}exp\left[-\frac{{\left(∆x_{ijk}-\lambda_{jk}∆\Lambda_{ijk}\right)}^{2}}{2\sigma_{jk}^{2}∆\Lambda_{ijk}}\right]$$

The corresponding log-likelihood equation is expressed as:

(14) $$lnL_{jk}\left(\theta \right)=-\frac{1}{2}\sum_{i}^{H_{jk}}\left[ln\left(2\pi \sigma_{jk}^{2}∆\Lambda_{ijk}\right)+\frac{{\left(∆x_{ijk}-\lambda_{jk}∆\Lambda_{ijk}\right)}^{2}}{\sigma_{jk}^{2}∆\Lambda_{ijk}}\right]$$

Notably, the accelerated degradation test is conducted based on the assumption that the degradation model remains identical but the parameter values vary.

As an example, the cumulative distribution functions $F_{k}\left(t_{k}\right)$ and $F_{h}\left(t_{h}\right)$ are separately under the stress of $T_{k}$ and $T_{h}$, respectively. As long as $F_{k}\left(t_{k}\right)=F_{h}\left(t_{h}\right)$, the acceleration factor is defined as $A_{k,h}=\frac{t_{h}}{t_{k}}$. Following the idea of Wang et al. [28], the acceleration factor relates to the stress level only if $A_{k,h}$ satisfies the following requirement:

(15) $$A_{k,h}={\left(\frac{\lambda_{h}}{\lambda_{k}}\right)}^{\frac{1}{\eta_{k}}}={\left(\frac{\sigma_{k}^{2}}{\sigma_{h}^{2}}\right)}^{\frac{1}{\eta_{k}}},\eta_{k}=\eta_{h}$$

In line with the non-linear Wiener process, this degradation mechanism is consistent and identical, which is determined by temperature variation under stress conditions. For this reason, the relationship between stress and its related parameters can be described using an acceleration model, i.e., the Arrhenius model [32,33]. Based on the constant acceleration factor, the Arrhenius equation for the degradation drift coefficient and diffusion coefficient is deduced as:

(16) $$\lambda \left(T\right)=e^{\left(\gamma_{1}-\frac{\gamma_{2}}{T}\right)}$$

(17) $$\sigma \left(T\right)=e^{\left(\gamma_{3}-\frac{0.5\gamma_{2}}{T}\right)}$$

where $\gamma_{1}$, $\gamma_{2}$ and $\gamma_{3}$ are constants to be calculated. Substituting Equations (16) and (17) into Equation (10), the Wiener–Arrhenius accelerated degradation model of PVS sensitivity is expressed as:

(18) $$x\left(t;T\right)=e^{\left(\gamma_{1}-\frac{\gamma_{2}}{T}\right)}t^{\eta }+e^{\left(\gamma_{3}-\frac{0.5\gamma_{2}}{T}\right)}B\left(t^{\eta }\right)$$

As mentioned above, each $∆x_{ijk}$ correlates to the normal distribution as:

(19) $$∆x_{ijk}\sim N\left(e^{\left(\gamma_{1}-\gamma_{2}/T_{k}\right)}∆\Lambda_{ijk},e^{\left({2\gamma }_{3}-\gamma_{2}/T_{k}\right)}∆\Lambda_{ijk}\right)$$

An unknown parameter set $\theta =\left(\gamma_{1},\gamma_{2},\gamma_{3},\eta \right)$ is thus established. Specifically, a likelihood function is developed by obtaining the degradation data for all thermal-stress conditions, which is:

(20) $$L\left(\theta \right)=\prod_{k}^{M}\prod_{j}^{N_{k}}\prod_{i}^{H_{jk}}\frac{1}{\sqrt{2\pi exp\left({2\gamma }_{3}-\gamma_{2}/T_{k}\right)∆\Lambda_{ijk}}}exp\left[-\frac{{\left(∆x_{ijk}-e^{\left(\gamma_{1}-\gamma_{2}/T_{k}\right)}∆\Lambda_{ijk}\right)}^{2}}{2e^{\left({2\gamma }_{3}-\gamma_{2}/T_{k}\right)}∆\Lambda_{ijk}}\right]$$

The corresponding log-likelihood equation is:

(21) $$lnL\left(\theta \right)=\sum_{k}^{M}\sum_{j}^{N_{k}}\sum_{i}^{H_{jk}}\left[-\frac{1}{2}\ln\left(2\pi \right)-\gamma_{3}+\gamma_{2}/2T_{k}-\frac{1}{2}\ln\left(∆\Lambda_{ijk}\right)-\frac{{\left(∆x_{ijk}-e^{\left(\gamma_{1}-\gamma_{2}/T_{k}\right)}∆\Lambda_{ijk}\right)}^{2}}{2e^{\left({2\gamma }_{3}-\gamma_{2}/T_{k}\right)}∆\Lambda_{ijk}}\right]$$

with the settling of $\hat{\theta }=\left({\hat{\gamma }}_{1},{\hat{\gamma }}_{2},{\hat{\gamma }}_{3},\hat{\eta }\right)$ using MLE, the AFCP-based Wiener–Arrhenius accelerated sensitivity degradation process is established.

### 4.2. Dynamic Error Correction

It is worth noting that the use of historical data can lead to inaccuracy in the sensitivity degradation model due to data deviation and discrepancy. Aiming at improving the prediction accuracy, the dynamic correction of the proposed model, based on the real-time sensitivity of the DT, is highlighted.

Assume now that the accelerated degradation model is calibrated at time $t_{k}$ with temperature T′, the degradation data $x_{k+1},x_{k+2},{\cdots ,x}_{k+m}$ at time $t_{k+1},t_{k+2},\cdots ,t_{k+m}$ is predicted using Equation (18). The predicted value in line with mathematical expectation can be:

(22) $${\hat{x}}_{k+b}=x_{k}+E\left[x\left(t_{k+b};T^{\prime }\right)-x\left(t_{k};T^{\prime }\right)\right]=x_{k}+e^{\left(\gamma_{1}-\frac{\gamma_{2}}{T^{\prime }}\right)}\left(t_{k+b}^{\eta }-t_{k}^{\eta }\right)$$

where b=1,2,⋯,m.

Moreover, the error between the predicted data and the measured data from time $t_{k+1}$ to $t_{k+m}$ is defined as:

(23) $$e_{k+b}={\hat{x}}_{k+b}-x_{k+b}$$

The error function $e\left(t\right)$ is derived by modeling the error sequence $e_{k+1:k+m}$. According to the physical property of predicted error, four error models can be adapted to the dynamic calibration, as listed in Table 1.

Distinctively, the error correction model is selected based on AIC (Akaike information criterion). We use the AIC value to weigh the model fitting effect and model complexity, which is computed as [35,36]

(24) $$AIC=2ln\left[L\left(D|M\right)\right]+2c$$

where $ln[L\left(D|M\right)]$ stands for the logarithmic maximum likelihood function, and c represents the number of unsettled parameters. The smaller the AIC value, the better the model fits the prediction error.

The selected error model is used as a supplementary to Equation (18). The calibrated sensitivity degradation from $t_{k+1}$ to $t_{k+m}$ under thermal stress T′ is given as:

(25) $$x\left(t;T^{\prime }\right)=e^{\left(\gamma_{1}-\frac{\gamma_{2}}{T^{\prime }}\right)}t^{\eta }+e^{\left(\gamma_{3}-\frac{0.5\gamma_{2}}{T^{\prime }}\right)}B\left(t^{\eta }\right)-e\left(t\right),t_{k+1}<t<t_{k+m}$$

where $x\left(t;T\prime \right)$ refers to the sensitivity degradation amount at time *t* under thermal stress T′. Along with the degradation process, the error correction is dynamically performed based on the DT data. As such, a more precise accelerated sensitivity degradation model is available using dynamic calibration.

### 4.3. Parameter Updating Using the Bayesian Method

The parameters of the original sensitivity degradation model are updated using the Bayesian method [37]. Substituting Equations (16) and (17) into Equation (25), accelerated sensitivity degradation is rewritten as:

(26) $$x\left(t;T^{\prime }\right)=\lambda t^{\eta }+\sigma B\left(t^{\eta }\right)-e\left(t\right),t_{k+1}<t<t_{k+m}$$

where $\lambda \sim N(\mu_{\lambda },\sigma_{\lambda }^{2})$, a of $e\left(t\right)$ correlates to $a\sim N\left(\mu_{a},\sigma_{a}^{2}\right)$, $\hat{\lambda }=e^{\left({\hat{\gamma }}_{1}-{\hat{\gamma }}_{2}/T\prime \right)},$ and $\hat{\sigma }=e^{\left({\hat{\gamma }}_{3}-0.5{\hat{\gamma }}_{2}/T\prime \right)}$. The posterior distribution of the parameters obtained using the Bayesian method is:

(27) $$p\left(\lambda ,a|x_{k+1:k+m}\right)\propto p\left(x_{k+1:k+m}|\lambda ,a\right)\pi_{k}\left(\lambda ,a\right)$$

where $\pi_{k}(\lambda ,a)$ is the joint prior distribution of λ and a with $\left(\lambda ,a)\sim MVN(\mu_{\lambda ,k},\mu_{a,k},\sigma_{\lambda ,k}^{2},\sigma_{a,k}^{2},\rho_{k}\right)$.

According to the conjugate property of the normal distribution, the joint posterior distribution of λ and a yields a bivariate normal distribution:

(28) $$\left(\lambda ,a\right)|x_{k+1:k+m}\sim MVN\left(\mu_{\lambda ,k+m},\mu_{a,k+m},\sigma_{\lambda ,k+m}^{2},\sigma_{a,k+m}^{2},\rho_{k+m}\right)$$

Conforming to the probability density function model of the bivariate normal distribution, we have:

(29) $$\begin{array}{c} p\left(\lambda ,a|x_{k+1:k+m}\right)\\ \propto exp\left[-\sum_{i=k+1}^{k+m}\frac{{\left(x_{i}-x_{i-1}+e\left(t_{i}\right)-e\left(t_{i-1}\right)-\lambda \left(t_{i}^{\eta }-t_{i-1}^{\eta }\right)\right)}^{2}}{2\sigma^{2}\left(t_{i}^{\eta }-t_{i-1}^{\eta }\right)}\right]\\ \times \;exp\left[-\frac{\frac{{\left(\lambda -\mu_{\lambda ,k+m}\right)}^{2}}{\sigma_{\lambda ,k+m}^{2}}-2\rho_{k+m}\frac{\left(\lambda -\mu_{\lambda ,k+m}\right)\left(a-\mu_{a,k+m}\right)}{\sigma_{\lambda ,k+m}\sigma_{a,k+m}}+\frac{{\left(a-\mu_{\lambda ,k+m}\right)}^{2}}{\sigma_{a,k+m}^{2}}}{2\left(1-\rho_{k+m}^{2}\right)}\right] \end{array}$$

Combining Equations (25) and (27), the posterior distribution parameters of λ and a in the error correction degradation model at time $t_{k+m}$ under T′ are expressed as $\left(\mu_{\lambda ,k+m},\mu_{a,k+m},\sigma_{\lambda ,k+m}^{2},\sigma_{a,k+m}^{2}\right)$. In addition, the hyperparameters $\left(\mu_{\lambda ,k},\mu_{a,k},\sigma_{\lambda ,k}^{2},\sigma_{a,k}^{2},\rho_{k}\right)$ in the joint prior distribution $\pi_{k}(\lambda ,a)$ can be determined using the expectation maximum (ME) method, whose optimal estimation is $\left({\hat{\mu }}_{\lambda ,k},{\hat{\mu }}_{a,k},{\hat{\sigma }}_{\lambda ,k}^{2},{\hat{\sigma }}_{a,k}^{2},{\hat{\rho }}_{k}\right)$.

### 4.4. RUL Prediction

With respect to the sensitivity degradation of PVS, the lifetime Life is the first hitting time (FHT) of failure threshold D, i.e.,

(30) $$Life=inf\left\{t:x\left(t\right)\geq D|x_{0}<D\right\}$$

Theoretically, RUL is defined as the span from a certain time to the time that the degradation amount first rises to the failure threshold. Under such thermal stress T′, the RUL $L_{k+m}$ at time $t_{k+m}$ derived from the corrected model is:

(31) $$L_{k+m}=inf\left\{l_{k}:x\left(t_{k+m}+l_{k+m}\right)\geq D|x\left(t_{k+m}\right)<D\right\}$$

The probability density function (PDF) of the lifetime can be simplified as [38]:

(32) $$f_{Life|\lambda ,a}\left(t|\lambda ,a\right)≅\frac{D+\lambda (\eta -1)t^{\eta }+e(t)-tde(t)/dt}{\sigma \sqrt{2\pi t^{3}}}e^{-\frac{{\left[D-\lambda t^{\eta }+e\left(t\right)\right]}^{2}}{2\sigma^{2}t}}$$

The degradation process under the current stress conditions is denoted as:

(33) $$Z\left(l_{k+s}\right)=x\left(t_{k+m}+l_{k+m}\right)-x\left(t_{k+m}\right)=\lambda {\left(t_{k+m}+l_{k+m}\right)}^{\eta }-\lambda t_{k+m}^{\eta }+\sigma B\left(l_{k+m}^{\eta }\right)-\Delta e\left(l_{k+m}\right)$$

with

(34) $$\Delta e\left(l_{k+m}\right)=e\left(t_{k+m}+l_{k+m}\right)-e\left(t_{k+m}\right)$$

The time for $Z\left(l_{k+m}\right)$ to first reach $D-x_{k+m}$ is the RUL, which is given by:

(35) $$\begin{array}{c} f_{L_{k+m}|\lambda ,a,x_{k+1:k+m}}\left(l_{k+m}|\lambda ,a,x_{k+1:k+m}\right)\\ ≅\frac{D-x_{k+m}+\Delta e\left(l_{k+m}\right)-l_{k+m}\frac{d\Delta e\left(l_{k+m}\right)}{dl_{k+m}}-\lambda {\left(t_{k+m}+l_{k+m}\right)}^{\eta }+\lambda t_{k+m}^{\eta }+\lambda \eta {l_{k+m}\left(t_{k+m}+l_{k+m}\right)}^{\eta -1}}{\sqrt{2\pi \sigma^{2}l_{k+m}^{3}}}\\ \times \;exp\left\{-\frac{{\left[D-x_{k+m}+\Delta e\left(l_{k+m}\right)-\lambda {\left(t_{k+m}+l_{k+m}\right)}^{\eta }+\lambda t_{k+m}^{\eta }\right]}^{2}}{2\sigma^{2}l_{k+m}}\right\} \end{array}$$

Subsequently, the remaining life $f_{L_{k+s}|x_{k+1:k+s}}\left(l_{k+s}|x_{k+1:k+s}\right)$ is obtained using the total probability theorem:

(36) $$f_{L_{k+m}|x_{k+1:k+m}}\left(l_{k+m}|x_{k+1:k+m}\right)=E_{a}\left[E_{\lambda |a}\left[f_{L_{k+m}|\lambda ,a,x_{k+1:k+m}}\left(l_{k+m}|\lambda ,a,x_{k+1:k+m}\right)\right]\right]$$

whose specific solution method is found in reference [39].

The point estimation of remaining life ${\hat{l}}_{k+s}$ is found by computing the median of Equation (25) [40], which is:

(37) $$Pr\left(L_{k+m}\leq {\hat{l}}_{k+m}|x_{k+1:k+m}\right)=0.5$$

For a constant 0<α<1, the $100\%\left(1-\alpha \right)$ confidence interval of the remaining life at time $t_{k+m}$ is given as ${(l}_{\alpha /2,k+m},l_{1-\alpha /2,k+m})$, which satisfies:

(38) $$Pr\left(L_{k+m}\leq l_{\alpha ,k+m}|x_{k+1:k+m}\right)=1-\alpha$$

## 5. Results and Discussion

### 5.1. Experiments Setup

**Figure 9:**
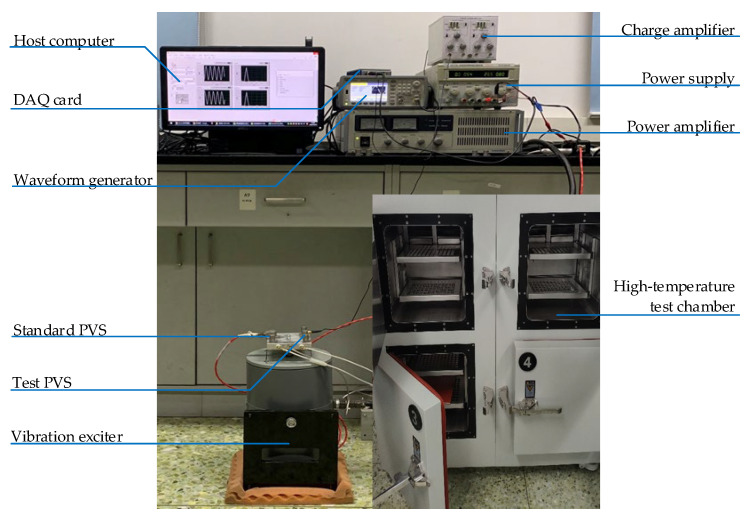
Experimental platform.

To validate the DT model of a PVS during the task of sensitivity degradation, a verification platform is established. A photograph of the test rig is shown in Figure 9.

During this test, a test PVS and a standard PVS are embedded in a vibration exciter for sensitivity degradation monitoring. The vibration signals are transformed through a charge amplifier, detected using a data acquisition (DAQ) card, and recorded with a host computer. We use a power supply as the electric source of the experimental system and a waveform generator for system debugging. In addition, a high-temperature test chamber is used to simulate the thermal stress condition. The specifications of each piece of equipment are given in Table 2.

Eight PVSs are randomly selected from the same batch and divided into four groups, namely, A, B, C, and D, for the sensitivity degradation test, which are labeled as (A1, A2), (B1, B2), (C1, C2), and (D1, D2), respectively. Aiming at revealing the effectiveness of our approach, the individual variation between the two samples in the same group is neglectable. The test procedures for all groups are presented as follows:

1. All PVSs are placed in the high-temperature test chamber and heated at a fixed rate;
2. The PVSs are removed from the high-temperature test chamber with a fixed cooling rate and embedded on the vibration exciter;
3. The acceleration is set at 100 Hz and 10 g, and the test PVSs’ function and sensitivity are set;
4. As long as a malfunction or sensitivity degradation reaches 20%, the test is terminated.

Notably, the temperature varies for different PVS groups, as listed in Table 3.

### 5.2. Experimental Results

**Figure 10:**
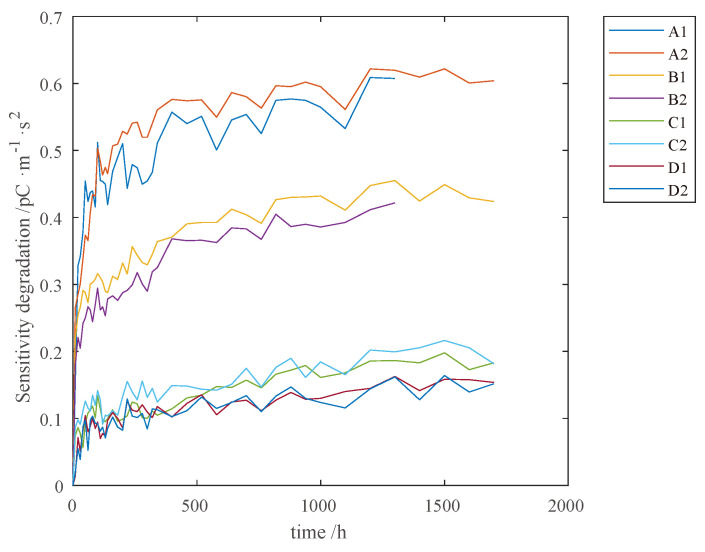
Sensitivity degradation of PVS samples.

The sensitivity degradation of distinctive PVSs is presented in Figure 10. The X-axis represents the test time, whilst the Y-axis stands for the variation in sensitivity. For each test sample, the degradation of sensitivity is performed in a non-monotonic process. By contrast, the degradation curves for PVSs in the same group are similar to each other due to the identical conditions during the test. One can observe that the higher the temperature, the faster the degradation rate, and thus, a greater degradation amount is accumulated.

### 5.3. Model Fitting and Verification

#### 5.3.1. Sensitivity Degradation Model Test

**Figure 11:**
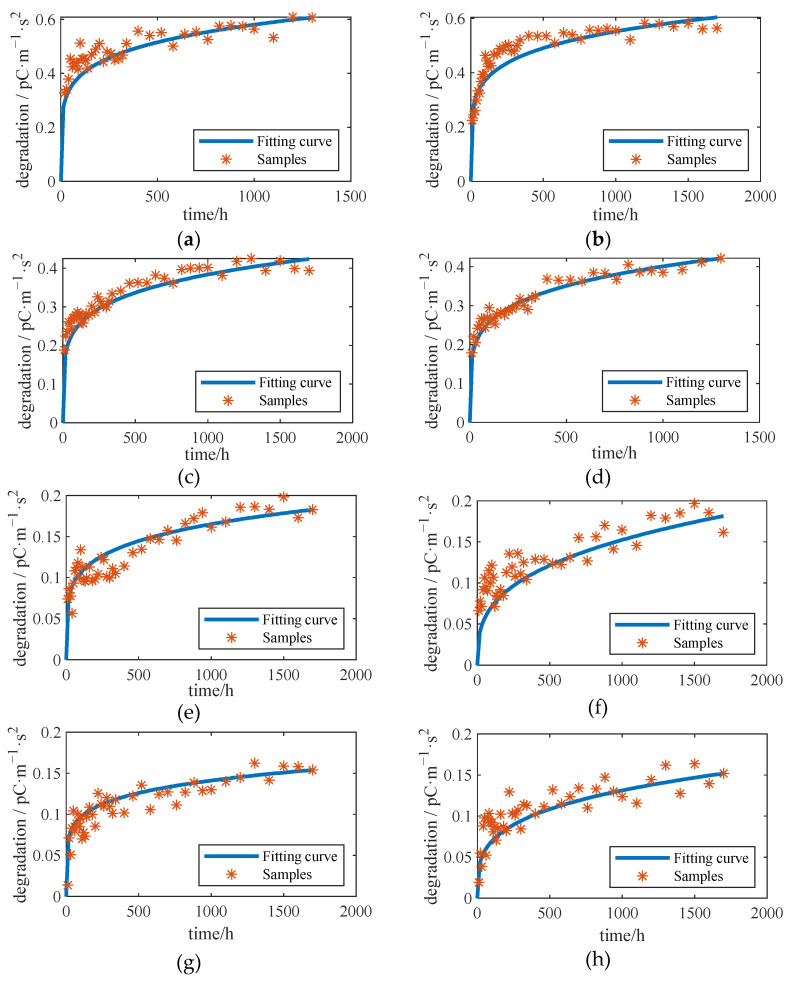
Sensitivity Degradation Model Fitting Results: (**a**) sample A1, (**b**) sample A2, (**c**) sample B1, (**d**) sample B2, (**e**) sample C1, (**f**) sample C2, (**g**) sample D1 and (**h**) sample D2.

As pointed out in the sensitivity degradation modeling, the degradation process is characterized by three parameters, i.e., λ, σ, and η. In line with the MLE method, the parameter fitting results at four diversified temperatures are listed in Table 4. We also report the sensitivity degradation trajectories in Figure 11.

For each sample, we have $∆{\hat{x}}_{ijk}\sim N\left({\hat{\lambda }}_{jk}∆\Lambda \left(t\right),{\hat{\sigma }}_{jk}^{2}∆\Lambda \left(t\right)\right),$ where $∆{\hat{x}}_{ijk}={\hat{x}}_{ijk}-{\hat{x}}_{(i-1)jk}$ and $∆\Lambda \left(t\right)=t_{ijk}^{\hat{\eta }}-t_{(i-1)jk}^{\hat{\eta }}$. Only if the fitting result is identical to this distribution can the degradation model be recognized as suitable. Accordingly, the hypothesis of $∆{\hat{x}}_{ijk}\sim N\left({\hat{\lambda }}_{jk}∆\Lambda \left(t\right),{\hat{\sigma }}_{jk}^{2}∆\Lambda \left(t\right)\right)$ is transformed into $\frac{∆{\hat{x}}_{ijk}-{\hat{\lambda }}_{jk}∆\Lambda \left(t\right)}{\sqrt{{\hat{\sigma }}_{jk}^{2}∆\Lambda \left(t\right)}}\sim N\left(0,1\right)$. Then, the K-S test is applied to investigate the standard normal distribution hypothesis. All the test *p*-values for the degradation samples exceed 0.05 (Table 5). The hypothesis at this stage is not rejected, confirming the feasibility of the proposed sensitivity degradation model.

Furthermore, the accelerated degradation is described using the parameter set ${\hat{\lambda }}_{k}$, ${\hat{\sigma }}_{k}$ and ${\hat{\eta }}_{k}$, which conforms to:

(39) $$\frac{{\hat{\lambda }}_{k}}{{\hat{\sigma }}_{k}^{2}}=\frac{{\hat{\lambda }}_{h}}{{\hat{\sigma }}_{h}^{2}},{\hat{\eta }}_{k}={\hat{\eta }}_{h}$$

Given that the degradation process is distinguished for each sample, the estimated values of the degradation parameters are randomly distributed even at the same stress level. The resolution of Equation (36) is transformed into the significance test of $\frac{{\hat{\lambda }}_{k}}{{\hat{\sigma }}_{k}^{2}}$ with $\frac{{\hat{\lambda }}_{h}}{{\hat{\sigma }}_{h}^{2}}$ and ${\hat{\eta }}_{k}$ with ${\hat{\eta }}_{h}$, respectively, and then an analysis of variance (ANOVA) is used. If the *p*-value from the ANOVA is smaller than 0.05, these two items are significantly different, and vice versa. Assuming that the parameters of the two samples fail to satisfy Equation (36), their degradation mechanisms are distinct. In this way, the degradation data under at least one stress condition is unfounded and cannot be applied to parameter estimation in the accelerated degradation model. As shown in Table 6, a temperature of 423.15 K is used for the hypothesis test against the degradation model under other conditions, respectively. The *p*-values calculated in the ANOVA analysis are reported.

**Figure 12:**
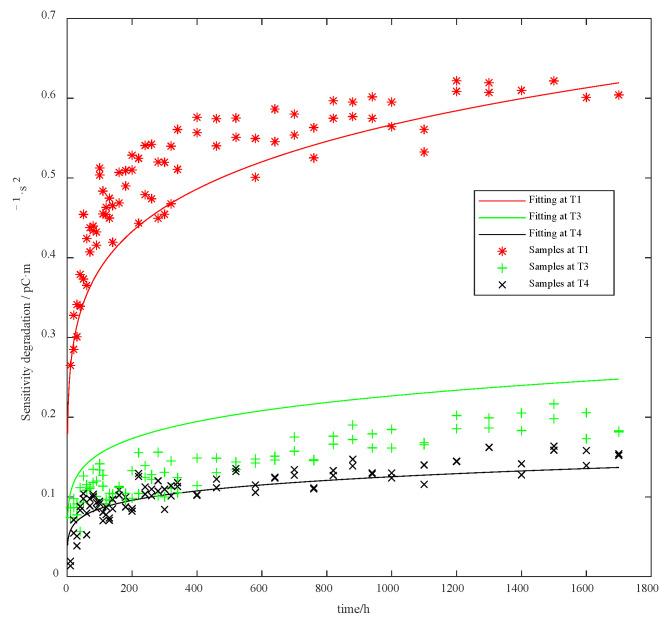
PVS sensitivity accelerated degradation model fitting results.

One can observe that the *p*-value between the parameters T2 and T4 is smaller than 0.04, indicating their degradation mechanisms are distinguished. With respect to the accelerated degradation model, the degradation data for T2 are removed. We thus use the three sets of data for parameter estimation. The Wiener–Arrhenius accelerated degradation model established from the estimated parameters is derived as follows:

(40) $$x\left(t;T\right)=e^{\left(7.3994-4499.3/T\right)}t^{0.1674}+e^{\left(2.6208-2249.7/T\right)}B\left(t^{0.1674}\right)$$

with ${\hat{\gamma }}_{1}=7.3994,{\hat{\gamma }}_{2}=4499.3,{\hat{\gamma }}_{3}=2.6208,$ and $\hat{\eta }=0.1674$, and the fitting results are shown in Figure 12.

According to Figure 12, our model generally covers the distribution of the given samples.

Likewise, for the sample in the accelerated degradation model, we have $∆{\hat{x}}_{ijk}\sim N\left(e^{\left({\hat{\gamma }}_{1}-{\hat{\gamma }}_{2}/T_{k}\right)}∆\Lambda_{ijk},e^{\left({2\hat{\gamma }}_{3}-{\hat{\gamma }}_{2}/T_{k}\right)}∆\Lambda_{ijk}\right)$, which can be transformed into $\frac{∆{\hat{x}}_{ijk}-e^{\left({\hat{\gamma }}_{1}-{\hat{\gamma }}_{2}/T_{k}\right)}∆\Lambda_{ijk}}{\sqrt{e^{\left({2\hat{\gamma }}_{3}-{\hat{\gamma }}_{2}/T_{k}\right)}∆\Lambda_{ijk}}}\sim N\left(0,1\right)$. Subsequently, the K-S test can be applied to verify the standard normal distribution of samples and, thus, the validity of the model.

**Figure 13:**
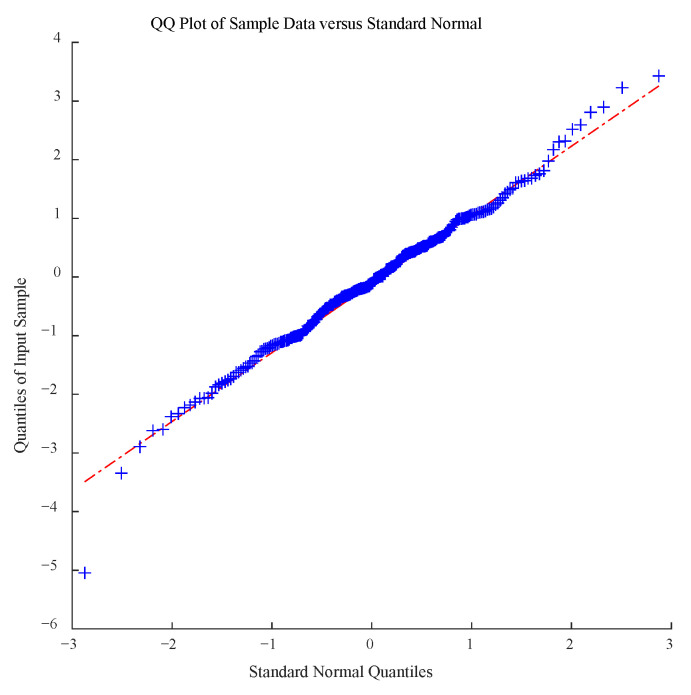
QQ-plot test results for the standard normal distribution.

The *p*-value of the K-S test is 0.0822, which is greater than 0.05, indicating that the model is suitable for describing accelerated sensitivity degradation. The degradation data tested using QQ-plot are shown in Figure 13.

#### 5.3.2. Dynamic Error Correction Analysis

The degradation test of sample E from the sample set is conducted at a temperature of 493.15 K. Based on the accelerated degradation model in Equation (40), we have:

(41) $$x\left(t\right)=e^{\left(7.3994-4499.3/493.15\right)}t^{0.1674}+e^{\left(2.6208-2249.7/493.15\right)}B\left(t^{0.1674}\right)$$

As presented in Equation (22), the degradation process of sample E can be modeled to predict the degradation data and the error $e_{t}$. Specifically, the first 100 h of data are used for error prediction while the model is corrected at the 100th h. According to Table 7, we obtain the AIC values of the error models in Table 1. The computation of each AIC value is given in Appendix B.

Based on AIC, M4 is selected for error fitting, which is:

(42) $$e_{1}\left(t\right)=0.0888sin\left(5.0994t+0.8960\right)+0.1270sin\left(1.2157t-2.7267\right)+0.0241sin\left(2.2777t-2.4995\right)+a_{1}$$

where $a_{1}\sim N(-0.1270,0.0010^{2})$.

**Figure 14:**
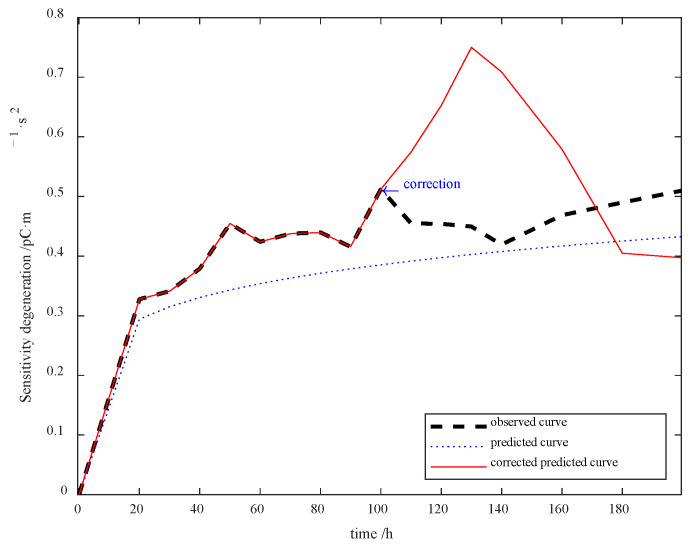
Revised sensitivity degradation prediction outcomes.

The revised sensitivity degradation error prediction is shown in Figure 14, which reveals the effectiveness of the AIC-based error correction scheme.

**Figure 15:**
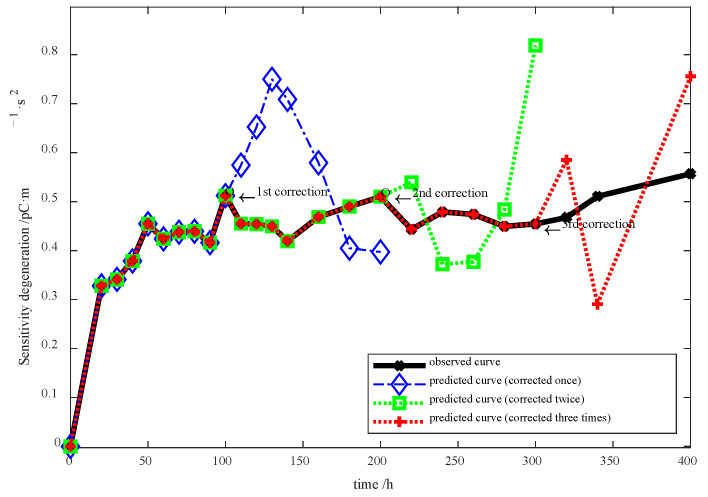
Sensitivity degradation prediction outcomes with different time error corrections.

Notwithstanding, the predicted error continues to increase after the first time model correction. For this reason, the 100 to 200 h degradation data are applied to correct the error at the 200th h, and so is that at the 300th h. In this experiment, three time error corrections were carried out, whose results are shown in Figure 15.

#### 5.3.3. RUL Prediction Results 

With respect to sample E, we can compute its degradation drift coefficient and diffusion coefficient using Equations (16) and (17) as $\lambda =e^{\left(\gamma_{1}-\gamma_{2}/T\right)}=0.1783$ and $\sigma =e^{\left(\gamma_{3}-0.5\gamma_{2}/T\right)}=0.1436$. The first 100 h of real-time degradation data are used for error correction. The parameters of the revised sensitivity degradation model are presented in Table 8.

In line with Equation (35), the revised sensitivity degradation model satisfies:

(43) $$\begin{array}{c} f_{L_{k+m}|\lambda ,a,x_{k+1:k+m}}\left(l_{k+m}|\lambda ,a,x_{k+1:k+m}\right)\\ ≅\frac{1}{\sqrt{2\pi \sigma^{2}l_{k+m}^{3}}}\{w-x_{k+m}+\sum_{j=1}^{n}b_{j}\sin\left(c_{j}l_{k+m}+c_{j}t_{k+m}+d_{j}\right)-\sum_{j=1}^{n}b_{j}\sin\left(c_{j}t_{k+m}+d_{j}\right) \\ -l_{k+m}\sum_{j=1}^{n}b_{j}c_{j}\cos\left(c_{j}l_{k+m}+c_{j}t_{k+m}+d_{j}\right)-\lambda {\left(t_{k+m}+l_{k+m}\right)}^{\eta }+\lambda t_{k+m}^{\eta }+\lambda \eta {l_{k+m}\left(t_{k+m}+l_{k+m}\right)}^{\eta -1}\}\\ \times \;exp\left\{-\frac{{\left[w-x_{k+m}+\sum_{j=1}^{n}b_{j}\sin\left(c_{j}l_{k+m}+c_{j}t_{k+m}+d_{j}\right)-\sum_{j=1}^{n}b_{j}\sin\left(c_{j}t_{k+m}+d_{j}\right)-\lambda {\left(t_{k+m}+l_{k+m}\right)}^{\eta }+\lambda t_{k+m}^{\eta }\right]}^{2}}{2\sigma^{2}l_{k+m}}\right\} \end{array}$$

Let $A=C-l_{k+m}\sum_{j=1}^{n}b_{j}c_{j}\cos\left(c_{j}l_{k+m}+c_{j}t_{k+m}+d_{j}\right)$, $B=D-{\eta l}_{k+m}{\left(t_{k+m}+l_{k+m}\right)}^{\eta -1}$, $C=w-x_{k+m}+\sum_{j=1}^{n}b_{j}\left[\sin\left(c_{j}l_{k+m}+c_{j}t_{k+m}+d_{j}\right)-\sin\left(c_{j}t_{k+m}+d_{j}\right)\right]$, and $D={\left(t_{k+m}+l_{k+m}\right)}^{\eta }-t_{k+m}^{\eta }$, $E=\sigma^{2}l_{k+m}$. Then, Equation (43) is converted to:

(44) $$f_{L_{k+m}|\lambda ,a,x_{k+1:k+m}}\left(l_{k+m}|\lambda ,a,x_{k+1:k+m}\right)≅\frac{A-B\lambda }{\sqrt{2\pi \sigma^{2}l_{k+m}^{3}}}\times exp\left[-\frac{{\left(C-D\lambda \right)}^{2}}{2E}\right]$$

Substituting Equation (44) into Equation (36), we have:

(45) $$\begin{array}{c} f_{L_{k+m}|x_{k+1:k+m}}\left(l_{k+m}|x_{k+1:k+m}\right)=E_{a}\left[E_{\lambda |a}\left[f_{L_{k+m}|\lambda ,a,x_{k+1:k+m}}\left(l_{k+m}|\lambda ,a,x_{k+1:k+m}\right)\right]\right]\\ =\frac{1}{\sqrt{2\pi \sigma^{2}l_{k+m}^{3}}}E_{\lambda }\left\{\left(A-B\lambda \right)\times exp\left[-\frac{{\left(C-D\lambda \right)}^{2}}{2E}\right]\right\}\\ =\frac{1}{\sqrt{2\pi l_{k+m}^{2}\left(D^{2}\sigma_{\lambda ,k+m}^{2}+E\right)}}\left(A-B\frac{CD\sigma_{\lambda ,k+m}^{2}+E\mu_{\lambda ,km}}{D^{2}\sigma_{\lambda ,k+m}^{2}+E}\right)\times exp\left[-\frac{{\left(C-D\mu_{\lambda ,k+m}\right)}^{2}}{2\left(D^{2}\sigma_{\lambda ,k+m}^{2}+E\right)}\right] \end{array}$$

**Figure 16:**
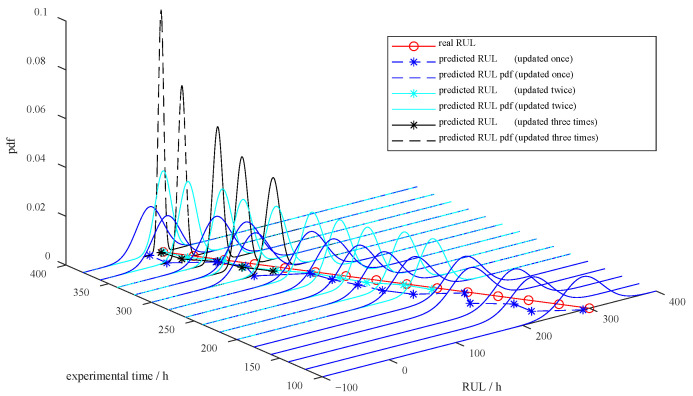
RUL prediction results with different times of model updates using real-time data.

The estimation of remaining life is computed as ${\hat{l}}_{k+s}=280\;h$. The 95% confidence interval for the predicted RUL at the 100th h is (230, 350) h, consistent with the measured 300th h RUL. The probability density function for the predicted RUL is shown in Figure 16. The probability density function narrows over time, indicating that the uncertainty in the RUL prediction results declines. In addition, with the increasing error correction times, an even higher RUL prediction accuracy is achieved. After three time error corrections, the prediction of RUL at the 300th h is 92 h, with a 95% confidence interval of (83, 104) h, which approaches the measured RUL of 100 h. In contrast, the RUL prediction outcome without error correction is 343 h at the 300th h, which has a considerable gap compared with the measured RUL of 100 h. In this way, it is reasonable to expect better-predicting performance of our model, as is the case.

### 5.4. Case Study

**Figure 17:**
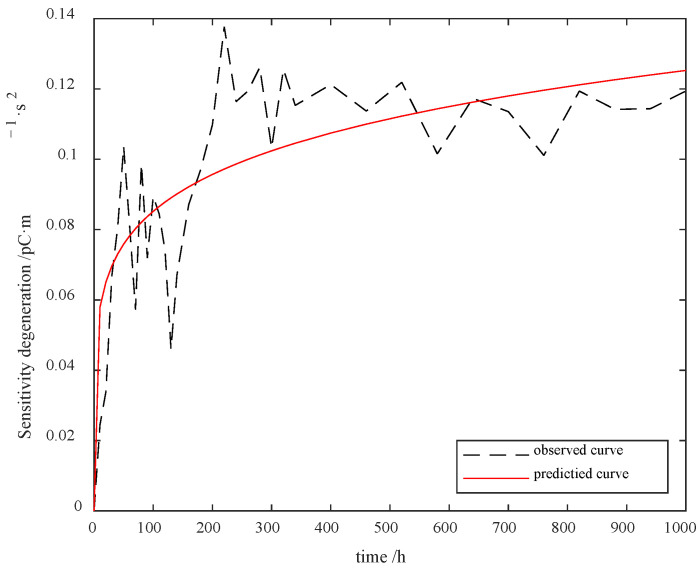
RUL prediction results compared with the observed results.

In line with the aforementioned procedures, a DT model of a PVS is established for RUL prediction. A set of PVS samples works at a temperature of 423.15 K, aiming at simulating the accelerated degradation process. Their working parameters can be sent to the DT model. Key parameters are obtained from the historical degradation data in the DT model, as listed in Table 9. A comparison of the predicted result and the observed result is shown in Figure 17.

The randomness of individual sensitivity degradation challenges precisely describes the degradation process with the basic prediction model. For this reason, the model modification is performed every 200 h by applying the DT real-time data to the sensitivity degradation analysis. The AIC values of the four error models are reported in Table 10. 

**Figure 18:**
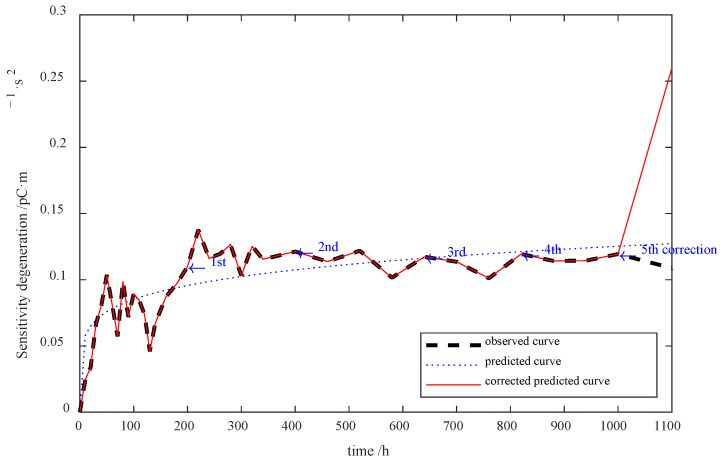
Sensitivity degradation of sample E.

The error model with the smallest AIC is used for error correction each time. Figure 18 shows the revised sensitivity degradation prediction results.

**Figure 19:**
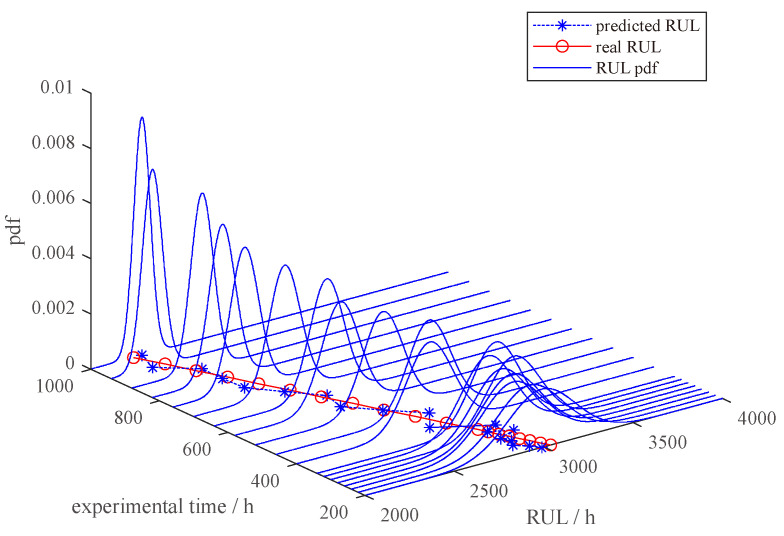
PVS RUL prediction results for sample E.

In such a manner, the model is further modified using the real-time data from DT. We obtain the 95% confidence interval for the RUL as (183.4, 214.7) h with the test processing set to 1000 h. The prediction is typically consistent with the observed RUL of 200 h in this experiment. The pdf of the RUL is illustrated in Figure 19. One can observe that the longer the interaction time with DT, the smaller the variance in the RUL prediction. As a result, the RUL prediction accuracy is substantially increased.

### 5.5. Discussion

In summary, the experimental results show that the proposed DT framework has distinctiveness in sensitivity degradation modeling of working PVS. Sensitivity degradation data are detected and generated in line with the DT framework, which is further applied to characterize the degradation process of a PVS under severe conditions. Moreover, the DT-based RUL prediction algorithm shows its superiority according to the experimental results. A Wiener–Arrhenius accelerated degradation model is built and verified using historical data and then revised. In addition, dynamic error correction and parameter updating are performed based on real-time degradation data. Specifically, the prediction method can effectively identify the RUL of PVS samples with different working hours. With the application of error correction, the prediction accuracy can be further improved in practical use.

Considering the multiple processes from model establishment to RUL prediction, no state-of-the-art methods are available for comparison. However, we compared our approach with the model without error correction. The experimental results for real PVS samples substantially highlight the effectiveness of our RUL prediction algorithm with high accuracy.

## 6. Conclusions

In this paper, a DT framework for RUL prediction of a PVS is proposed. To start with, on the basis of a five-dimensional model, the establishment of the DT model in five dimensions is analyzed, with a focus on signal modeling, structure simplification, and RUL prediction in *VE*. Furthermore, six modules, together with their relationship within the DT framework, are depicted. In the scope of RUL prediction, an accelerated degradation model that exploits historical degradation data is built. In addition, methods for error correction and parameter adjustment of the degradation model are put forward. Experiments are carried out on PVS samples to predict the RUL based on real-time degradation data. The experimental results reveal that an accurate RUL prediction result can be achieved in practical use. The following conclusions can be drawn:

(1) The DT framework comprehensively characterizes the structure and working principle of PVSs, precisely performing the sensitivity degradation process. Although onboard degradation data are absent, the operation data can be measured and generated based on the DT model, which is further applied to RUL prediction.
(2) Considering the high-temperature condition, a Wiener–Arrhenius accelerated degradation model is built using historical degradation data from DT. The proposed model is optimized with error correction using the AIC criterion and parameter updating using the Bayesian method, based on which the RUL is thus computed.
(3) Experiments on real PVS samples demonstrate the technical efficacy of our model. A RUL prediction is carried out with the integration of real-time degradation data. Compared with the measured RUL, the prediction result is obtained with a high accuracy.

## Figures and Tables

**Table 1:** Error Prediction Models [34].

Model	Function
M1	$e\left(t\right)=e\left(0\right)+a\int_{0}^{t}bexp(bt)dt$ *
M2	$e\left(t\right)=e\left(0\right)+a\int_{0}^{t}bt^{b-1}dt$ *
M3	$e\left(t\right)=b_{1}+\cdots +b_{p}t^{p}+a$ *
M4	$e\left(t\right)=\sum_{1}^{n}b_{i}sin(c_{i}t+d_{i})+a$ *

* $e\left(0\right)$ is the initial value; a denotes the random characteristics of error and correlates to the normal distribution $a\sim N\left(\mu_{a},\sigma_{a}^{2}\right)$; and other parameters are constants.

**Table 2:** Test equipment specifications.

Device	Parameters
High-temperature test chamber	LIGAO HF-100FN, 300 K~600 K
Vibration exciter	SINOCERA JZK-20, 200 N, 30 g
Power supply	SINOCERA YE5874, 810 W
Waveform generator	KEYSIGHT 33500B, 30 MHz, 5 V
Standard VPS	Endevco 6222S-20A, 200 mV/g
Charge amplifier	Endevco 2777A-10-10, 10 Hz~10 kHz
Host computer	ThinkStation P350, Intel i7 11700
Data acquisition card	NI Compact DAQ 9232, 3 channel, 102.4 kS/s/ch

**Table 3:** Test temperature.

Sensor Number	Temperature
A1 and A2	493.15 K
B1 and B2	473.15 K
C1 and C2	448.15 K
D1 and D2	423.15 K

**Table 4:** Sensitivity Degradation Model Parameter Fitting.

Stress	Sample	${\hat{\sigma }}_{k}$	${\hat{\lambda }}_{k}$	${\hat{\eta }}_{k}$
493.15 K	A1	0.1706	0.1773	0.1717
	A2	0.1071	0.1701	0.1704
473.15 K	B1	0.0722	0.1047	0.1881
	B2	0.0724	0.1061	0.1925
448.15 K	C1	0.0597	0.0440	0.1913
	C2	0.0424	0.0156	0.3296
423.15 K	D1	0.0794	0.0454	0.1641
	D2	0.0589	0.0196	0.2757

**Table 5:** Sensitivity degradation model verification based on the K-S test.

Sample	A1	A2	B1	B2	C1	C2	D1	D2
*p*-value	0.6023	0.3200	0.2104	0.5694	0.5551	0.7879	0.5114	0.9578
outcome	Accepted	Accepted	Accepted	Accepted	Accepted	Accepted	Accepted	Accepted

**Table 6:** ANOVA-calculated *p*-values of parameters under different conditions.

Working Condition	$T_{1},T_{4}$*	$T_{2},T_{4}$*	$T_{3},T_{4}$*
$\frac{{\hat{\lambda }}_{k}}{{\hat{\sigma }}_{k}^{2}},\frac{{\hat{\lambda }}_{h}}{{\hat{\sigma }}_{h}^{2}}$	0.4588	0.0033	0.1762
${\hat{\eta }}_{k},{\hat{\eta }}_{h}$	0.4743	0.6494	0.6922

* T1 = 493.15 K, T2 = 473.15 K, T3 = 448.15 K, T4 = 423.15 K.

**Table 7:** AIC value of four error models.

Error Model	M1	M2	M3	M4
AIC	−52.2544	−51.8594	−49.6261 (*p* = 1)	−101.7984 (*p* = 3)

**Table 8:** Parameters of the revised sensitivity degradation model.

Parameter	${\hat{\mu }}_{\lambda ,k}$	${\hat{\sigma }}_{\lambda ,k}^{2}$	${\hat{\mu }}_{a,k}$	${\hat{\sigma }}_{a,k}^{2}$	${\hat{\rho }}_{k}$	$\hat{\sigma }$	$\hat{\eta }$	$\hat{b}$
Value	0.2585	0.0351^2^	−0.1270	0.0010^2^	0.0090	0.0221	0.0762	b1, b2, b3: 0.1000, 0.0619, 0.0247 c1, c2, c3: 0.6633, 1.3372, −0.3959 d1, d2, d3: 0.2365, 0.5675, −0.4238

**Table 9:** Sensitivity degradation model parameters.

DT Model Parameter	${\hat{\gamma }}_{1}$	${\hat{\gamma }}_{2}$	${\hat{\gamma }}_{3}$	$\hat{\eta }$	*T*
Value	7.3994	4499.3	2.6208	0.1674	423.15

**Table 10:** AIC value of four error models.

Error Model	M1	M2	M3	M4
AIC at 200 h	−70.9	−73.5	−75.0	−124.0
AIC at 400 h	−46.9	−47.8	−61.7	−93.8
AIC at 600 h	−9.4	−9.6	−2.2	−31.4
AIC at 800 h	−11.9	−11.9	−11.8	−21.4
AIC at 1000 h	−11.94	−12.62	−11.04	−45.26

## Data Availability

The data are available from the authors upon request.

## Appendix A

### Appendix A.1. Structure of a Simplified Shear PVS

In practice, a vibration is delivered from the base to the piezoelectric strip and reaches the mass block, which sets the foundation for further analysis. In this way, these three components are preserved with only the structural details simplified. For model optimization, the modal analysis of a shear-type PVS is carried out by setting a threshold of natural frequency offset less than 1%, as listed in Table A1.

**Table A1:** Component simplification of a shear-type PVS.

Simplified Components	Natural Frequency/Hz	Deviation/%
-	7158.0	-
Insulation strip	7158.8	0.01
Insulation sleeve	7165.7	0.11
Conducting strip	7219.2	0.85
Connector	7157.1	−0.01
Cover	7158.1	0.00
Nut	7034.6	−1.72
Bolt	7386.7	3.20
Insulation strip, insulation sleeve, conducting strip, connector and cover	7226.9	0.96

According to the modal analysis results, components with smaller sizes and masses make minor contributions to the natural frequency, conforming to the mechanics of materials theory. One can observe that five types of components are maintained in a shear-type PVS, which are a base, piezoelectric strips, mass blocks, nuts, and a pre-load screw. The structural details of these components, especially the mounting, connecting, and wiring fixtures, are further analyzed for simplification, as listed in Table A2. With the preservation of the nut assembly fixture, the simplified structure of a shear-type PVS is confirmed.

**Table A2:** Assembly details of a simplified a shear-type PVS.

Simplified Structure	Natural Frequency/Hz	Deviation/%
-	7158.0	-
Base wiring hole	7161.6	0.05
Connector–base connection	7154.4	−0.05
Base mounting hole	7134.0	−0.34
Housing–base connection	7155.9	−0.03
Nut chamfer and wedge cut	6982.4	−2.45
Base wiring hole, connector–base connection, base mounting hole, and housing–base connection	7128.2	−0.42

### Appendix A.2. Structure of a Simplified Compression-type PVS

Likewise, we obtain a simplified structure of compression-type PVS by retaining the base, the piezoelectric strips, the conducting strips, the seismic mass, and the pre-load screw (Table A3) while ablating the assembly details (Table A4).

**Table A3:** Component simplification of a compression-type PVS.

Simplified Components	Natural Frequency/Hz	Deviation/%
-	13,253	-
Connector	13,236	−0.13
Housing	13,252	−0.01
Insulation strip	13,253	0.00
Conducting strip	19,542	47.45
Connector, housing, and insulation strip	13,227	−0.20

**Table A4:** Assembly details of a simplified compression-type PVS.

Simplified Structure	Natural Frequency/Hz	Deviation/%
-	13,253	-
Base mounting hole	13,237	−0.12
Connector–base connection	13,236	−0.13
Screw mounting hole	13,236	−0.13
Bottom mounting hole, connector–base connection, and screw mounting hole	13,237	−0.12

## Appendix B

### Appendix B.1. Error Model 1

For $e\left(t_{i}\right)=e\left(0\right)+a\int_{0}^{t_{i}}bexp(bt)dt=a\left(e^{bt_{i}}-1\right)$ with $a\sim N\left(\mu_{a},\sigma_{a}^{2}\right)$, we have $e\left(t_{i}\right)\sim N\left(\mu_{a}\left(e^{bt_{i}}-1\right),\sigma_{a}^{2}{\left(e^{bt_{i}}-1\right)}^{2}\right)$. The log-likelihood function is established as:

(A1) $${lnL}_{1}=-\frac{1}{2}\sum_{i=1}^{N}\left[ln\left(\sigma_{a}^{2}{\left(e^{bt_{i}}-1\right)}^{2}\right)+\frac{{\left(e\left(t_{i}\right)-\mu_{a}\left(e^{bt_{i}}-1\right)\right)}^{2}}{\sigma_{a}^{2}{\left(e^{bt_{i}}-1\right)}^{2}}\right]$$

The values of $\mu_{a}$, $\sigma_{a},$ and b are computed using MLE. The outcome of ${AIC}_{1}$ is derived from Equation (24).

### Appendix B.2. Error Model 2

For $e\left(t_{i}\right)=e\left(0\right)+a\int_{0}^{t}bt^{b-1}dt=a{t_{i}}^{b}$ with $a\sim N\left(\mu_{a},\sigma_{a}^{2}\right)$, we have $e\left(t_{i}\right)\sim N\left(\mu_{a}{t_{i}}^{b},\sigma_{a}^{2}{t_{i}}^{2b}\right)$. The log-likelihood function is established as:

(A2) $${lnL}_{2}=-\frac{1}{2}\sum_{i=1}^{N}\left[ln\left(\sigma_{a}^{2}{t_{i}}^{2b}\right)+\frac{{\left(e\left(t_{i}\right)-\mu_{a}{t_{i}}^{b}\right)}^{2}}{\sigma_{a}^{2}{t_{i}}^{2b}}\right]$$

The values of $\mu_{a}$, $\sigma_{a},$ and b are computed using MLE. The outcome of ${AIC}_{2}$ is derived from Equation (24).

### Appendix B.3. Error Model 3

For $e\left(t_{i}\right)=e\left(0\right)+b_{1}t_{i}+\cdots +b_{p}t^{p}+a=b_{1}t_{i}+\cdots +b_{p}t^{p}+a$ with $a\sim N\left(\mu_{a},\sigma_{a}^{2}\right)$, we have $e\left(t_{i}\right)\sim N\left({\mu_{a}+b}_{1}t_{i}+\cdots +b_{p}t^{p},\sigma_{a}^{2}\right)$. The log-likelihood function is established as:

(A3) $${lnL}_{3}=-\frac{1}{2}\sum_{i=1}^{N}\left[ln\left(\sigma_{a}^{2}\right)+\frac{{\left(e\left(t_{i}\right)-b_{1}t_{i}-\cdots -b_{p}{t_{i}}^{p}-\mu_{a}\right)}^{2}}{\sigma_{a}^{2}}\right]$$

The values of $\mu_{a}$, $\sigma_{a},$ and $b_{1}$, $b_{2}$, …, $b_{p}$ are computed using MLE at different positive integers p. The outcomes of the AIC with different p are derived from Equation (24), the maximum of which is taken as ${AIC}_{3}$.

### Appendix B.4. Error Model 4

For $e\left(t_{i}\right)=\sum_{j=1}^{p}b_{j}sin(c_{j}t+d_{j})+a$ with $a\sim N\left(\mu_{a},\sigma_{a}^{2}\right)$, we have $e\left(t_{i}\right)\sim N\left(\sum_{j=1}^{p}b_{j}sin(c_{j}t+d_{j})+\mu_{a},\sigma_{a}^{2}\right)$. The log-likelihood function is established as:

(A4) $${lnL}_{4}=-\frac{1}{2}\sum_{i=1}^{N}\left[ln\left(\sigma_{a}^{2}\right)+\frac{{\left(e\left(t_{i}\right)-\sum_{j=1}^{p}b_{j}sin(c_{j}t+d_{j})-\mu_{a}\right)}^{2}}{\sigma_{a}^{2}}\right]$$

The values of $\mu_{a}$, $\sigma_{a}$, $b_{1}$, $b_{2}$, …, $b_{p}$, $c_{1}$, $c_{2}$, …, $c_{p,}$ and $d_{1}$, $d_{2}$, …,$d_{p}$ are computed using MLE at different positive integers p. The outcomes of the AIC with different p are derived from Equation (24), the maximum of which is taken as ${AIC}_{4}$.
